# Assessing Serum Neurofilament Light Chain in Hereditary Transthyretin Amyloidosis: Direct Comparison of Three Immunoassays

**DOI:** 10.3390/jcm15041584

**Published:** 2026-02-18

**Authors:** Milou Berends, Johan Bijzet, Suzanne Arends, Elisabeth Brouwer, Charlotte E. Teunissen, Sjors G. J. G. in ’t Veld, Reinold O. B. Gans, Bouke P. C. Hazenberg, Paul A. van der Zwaag, Hans L. A. Nienhuis, Bart-Jan Kroesen

**Affiliations:** 1 Department of Internal Medicine, University Medical Center Groningen, 9713 GZ Groningen, The Netherlandsb.j.kroesen@umcg.nl (B.-J.K.); 2 Amyloidosis Center of Expertise, University Medical Center Groningen, 9713 GZ Groningen, The Netherlands; 3 Department of Laboratory Medicine, University Medical Center Groningen, 9713 GZ Groningen, The Netherlands; 4 Department of Rheumatology & Clinical Immunology, University Medical Center Groningen, 9713 GZ Groningen, The Netherlands; 5 Department of Laboratory Medicine, Amsterdam Neuroscience, Amsterdam UMC location Vrije Universiteit Amsterdam, 1081 HV Amsterdam, The Netherlands; 6 Department of Genetics, University Medical Center Groningen, 9713 GZ Groningen, The Netherlands

**Keywords:** ATTRv amyloidosis, enzyme-linked immunosorbent assay, Meso Scale Discovery R-PLEX assay, neurofilament light chain, single-molecule array assay

## Abstract

**Background/Objectives**: Serum neurofilament light chain (sNfL) is an early and sensitive biomarker of polyneuropathy. This study compared the UmanDiagnostics enzyme-linked immunosorbent assay (ELISA), and Meso Scale Discovery (MSD) R-PLEX assay with the current gold-standard single-molecule array (Simoa) assay for sNfL measurement. **Methods**: sNfL levels were measured with Simoa, ELISA, and MSD R-PLEX in 330 serum samples from 73 individuals with a pathogenic transthyretin gene variant (*TTR*v) and in 165 healthy controls (HC) with ELISA and MSD R-PLEX. **Results**: Median sNfL levels, assessed in serum samples from *TTRv* individuals, differed across all assays (all *p* < 0.001). Passing–Bablok regression slopes were 1.01 (Simoa–ELISA), 1.00 (Simoa–MSD R-PLEX), and 1.02 (MSD R-PLEX-ELISA), with very strong correlations (all r > 0.8). Bland–Altman analysis showed mean differences of 0.1 ± 0.2 pg/mL (Simoa–ELISA), 0.7 ± 0.1 pg/mL (Simoa–MSD R-PLEX), and −0.6 ± 0.2 pg/mL (MSD R-PLEX-ELISA). In HC, sNfL levels positively correlated with age. Z-score normalization allowed for inter-assay comparison. **Conclusions**: The ELISA and MSD R-PLEX assays provide suitable alternatives for the Simoa assay to measure sNfL levels in carriers of a pathogenic *TTR*-gene variant. The differences in concentrations defined by the assays directly relate to the internal standard provided with the assays.

## 1. Introduction

Hereditary transthyretin (ATTRv) amyloidosis is caused by pathogenic variants in the gene encoding the transthyretin (TTR) protein [1]. Mutated TTR monomers are prone to become misfolded and, as a consequence, aggregate and deposit as amyloid fibrils in various tissues and organs, including the nervous system and heart, leading to loss of function of these organs [2,3]. More than 150 pathogenic *TTR*-gene variants (*TTR*v) have been described [4,5]. The clinical presentation is variable, but peripheral and autonomic neuropathy are frequent among the first disease manifestations [6]. Once started, the disease course is progressive and, if left untreated, lethal after 6–12 years [7,8,9]. Therefore, early diagnosis, enabling the early initiation of treatment, is critical for achieving the best possible outcome.

Axonal nerve damage results in the release of neurofilament light chain (NfL) from neurons and increased cerebrospinal and serum NfL (sNfL) levels [10]. We recently found sNfL to be a sensitive biomarker for polyneuropathy in patients with ATTRv amyloidosis. Moreover, a rise in sNfL precedes the onset of polyneuropathy symptoms [11,12]. Furthermore, treatment modalities that effectively stabilize the TTR protein or control *TTR*-gene expression have shown to correlate with stabilized or decreased sNfL levels, respectively [13]. However, the routine measurement of sNfL in clinical diagnostic setting is still limited due to the restricted availability of standardized assays across laboratories.

The Quanterix single-molecule array (Simoa) NfL assay is the current gold standard for measuring NfL in serum or plasma in research settings [14]. The Simoa assay is a time-lapsed, fluorescence-based digital immunoassay that uses microfluidics to compartmentalize immunocomplexes into femtoliter-sized chambers, enabling single-molecule detection with high sensitivity [15,16]. Another assay to quantify NfL levels is the Meso Scale Discovery (MSD) R-PLEX Human Neurofilament L assay. The MSD R-PLEX is an immunoassay that, similar to the Simoa assay, is highly sensitive but uses an electrochemiluminescence (ECL) technique to detect the analyte. Specifically, the assay utilizes a SULFO-TAG ECL label that emits light upon electrochemical stimulation initiated at the electrode surfaces of microplates [17]. Both assays provide precise and specific protein quantification at very low, pg/mL, concentrations. Both assay formats are not generally available in clinical diagnostic laboratories. Recently, an updated version of a previously described sNfL enzyme-linked immunosorbent assay (ELISA) was introduced by Uman Diagnostics. This ELISA uses the same antibodies as the Simoa assay to quantify sNfL levels [18]. While the previous version [19] had relatively low sensitivity, as noted in a personal communication with Kuhle et al. and discussed by Pafiti et al. [20], the updated version has a detection limit that is similar to the Simoa assay. Moreover, the ELISA format is common in most clinical diagnostic laboratories and, as such, easy to implement in diagnostic settings.

The availability of different validated assays will facilitate the widespread implementation of sNfL in routine diagnostic practice. Moreover, a diverse and unbiased diagnostic landscape is built upon the use of different assay platforms. However, to ensure the accurate interpretation and comparison of sNfL results obtained using different assays, understanding individual assay characteristics and inter-assay variability is essential.

In this study, we compared the results of the ELISA and MSD R-PLEX assays with the current gold-standard Simoa assay to measure sNfL levels in carriers of a pathogenic *TTR-*gene variant and healthy controls (HC). This study aimed to determine whether the ELISA and the MSD R-PLEX assays are suitable alternatives for the Simoa assay, allowing for the implementation of sNfL measurement in a clinical diagnostic setting.

## 2. Materials and Methods

### 2.1. Study Design

In this retrospective study, we used serum samples collected from carriers of a pathogenic *TTR-*gene variant that had been collected for routine patient care in the ‘AmyloidLines’ biobank in the period between January 2000 and December 2021. Another cohort with samples from HC collected between November 2006 and March 2016 was added [21].

Individuals carrying a pathogenic *TTR*-gene variant, both asymptomatic and symptomatic (for definitions see Table S1), were further referred to as the ‘study population’ [11,12]. Data included age, sex, and kidney function (estimated glomerular filtration rate (eGFR)) at the time of sNfL sampling. For HC, only data about age and sex at the time of sNfL sampling were collected.

All procedures were compliant with the Declaration of Helsinki. The study was approved by the institutional ethical review board of the University Medical Center Groningen (UMCG) (AmyloidLines registration number 17471 and Immune aging in health and disease METC number 2012.375) [21].

### 2.2. Study Population

We analyzed sNfL levels in 330 samples collected at multiple time points from 73 pathogenic *TTR*-gene variant carriers using the Simoa, ELISA, and MSD R-PLEX assays to evaluate assay agreement.

Additionally, 165 samples from unique HC were included as a healthy reference cohort. Health status was evaluated by taking medical history, a physical examination, blood testing and urinalysis, as described previously [21]. In this cohort, sNfL levels were measured using the ELISA and MSD R-PLEX assays.

### 2.3. Serum Handling and Quantification of sNfL

Blood samples were drawn by venipuncture at the UMCG outpatient clinic, centrifuged at 2700 rpm for ten minutes at room temperature, and stored for an hour at −20 °C. Subsequent storage at −80 °C for later use was performed within six months.

Measurement of sNfL was performed using the Quanterix Simoa NF-light Kit (Simoa HD-X) technology (Quanterix Corp.; Billerica, MA, USA) [22], Quanterix-Uman Diagnostics NF-light™ Serum ELISA (Tecan Benelux, Giessen, The Netherlands) [18], and MSD R-PLEX Human Neurofilament L assay (Meso-Scale Discovery; Rockville, MD, USA) [23]. The ELISA and MSD R-PLEX assays were performed at the department of Medical Immunology (UMCG) according to the manufacturer’s instructions. The Simoa assay was performed at the Neurochemistry laboratory of the Amsterdam University Medical Center location VUmc, according to the manufacturer’s instructions. Measurements were performed by certified technicians who were blinded to clinical information.

### 2.4. Internal Standard NfL

To compare the differences observed in sNfL values between the different assays, internal standards from the Simoa and MSD R-PLEX assays were cross-measured using the ELISA and MSD R-PLEX assays. For this purpose, the internal standards supplied with each assay were serially diluted and analyzed across the different assays.

### 2.5. Statistical Analysis

Categorical variables were presented as absolute numbers and percentages, normally distributed variables as means and standard deviations (SD), and non-normally distributed variables as medians and interquartile range (p25–p75). Normality plots with tests were performed to assess the normality of the data. Corresponding sNfL levels measured by the three assays were compared using the Friedman test with post hoc Wilcoxon signed rank test.

Intra- and inter-assay coefficient of variation (CV) was evaluated with three control serum samples for the ELISA and MSD R-PLEX assays [24]. The limit of detection (LOD) and limit of quantitation (LOQ) were calculated according to Cantwell et al. [25].

Method agreement and proportional bias between the assay methods were estimated using Passing–Bablok regression analysis on log-transformed sNfL levels to meet the assumption of a linear relationship between the assays. The regression equation Log10(Y)=a+b∗Log10(X) revealed a constant (intercept, *a*) and proportional (slope, *b*) difference. The Pearson correlation coefficient was calculated to assess the relationship between assays. Correlation strength was interpreted as poor (0.0–0.2), fair (>0.2–0.4), moderate (>0.4–0.6), good (>0.6–0.8), or excellent (>0.8–1.0) [26].

Bland–Altman analysis was used to quantify the mean difference and 95% limits of agreement to provide insight in the systematic bias and random error between the different assay methods. Log-transformed sNfL levels were used to meet the assumption of normally distributed differences. The acceptance limits based on the inherent imprecision of the assay methods were calculated as $\sqrt{\frac{intra\;-\;assay\;CV_{A}^{2}}{n}\;+\;\frac{intra\;-\;assay\;CV_{B}^{2}}{n}}$, with A and B corresponding to the assays used and *n* to the number of samples used for calculating the intra-assay CV (further explained in File S1) [27].

In HC, differences in sNfL levels and age between males and females were tested by the Mann–Whitney test. Corresponding sNfL levels measured by the ELISA and MSD R-PLEX assays were compared using the Wilcoxon signed rank test. sNfL levels measured using the ELISA and the MSD R-PLEX assays were tested for correlation with age using Spearman’s rho test.

Z-scores were calculated to enable standardized comparison between the assays. To meet the assumption of normal distribution, Z-scores were calculated from log-transformed sNfL levels. Mean and SD were calculated for all three assays. Z-scores were calculated as: $\frac{sNfL\;level\;individual\;patient\;-\;mean\;sNfL\;level\;study\;population}{SD\;study\;population}$. Z-scores measured by the three assays were compared using a paired *t*-test.

A two-sided Mann–Whitney test was used to compare sNfL levels between different study groups (*TTR*v carriers, asymptomatic ATTRv patients and symptomatic ATTRv patients).

The primary objective of this study was to compare different assays rather than to draw inferences at the patient level. Therefore, no formal sensitivity analyses were performed to account for repeated measurements within individuals.

Statistical analysis was performed using IBM SPSS Statistics version 28.0 (IBM Corp, Armonk, NY, USA) and R software version 4.4.3. Graphs were generated with GraphPad Prism version 8.4.2 (GraphPad Software, La Jolla, CA, USA). *p*-values < 0.05 were considered statistically significant.

## 3. Results

### 3.1. Assay Characteristics

The ELISA mean intra- and inter-assay CV were 3.6% and 3.8%, respectively. The LOD and LOQ were 1.8 pg/mL and 2.0 pg/mL, respectively. For the MSD R-PLEX assay, mean intra- and inter-assay CV were 6.8% and 8.0%, respectively. The LOD and LOQ were 8.4 pg/mL and 9.8 pg/mL, respectively.

### 3.2. Baseline Characteristics Study Population and Healthy Controls

In the study population, 73 individuals were included, with one to 27 samples per individual collected, resulting in a total of 330 samples. Mean age was 53 ± 14 years and females constituted 56% of the total study population (Table 1). The most common *TTR*-gene variant was Val30Met (p.Val50Met) (Table S2). No correlation was found between eGFR and sNfL for either of the assays.

The mean age of the HC cohort was 51 ± 17 years and females constituted 62% of the total group (Table 1). Males were older than females (mean 55 ± 16 versus 48 ± 18; *p* = 0.01). No significant differences were observed in median sNfL levels per gender per assay. sNfL levels positively correlated with age (ELISA r = 0.56 (95% confidence interval (CI) (0.4–0.7), and MSD R-PLEX assay r = 0.50 (95% CI (0.3–0.6)) (both *p* < 0.001) (Figure S1).

### 3.3. sNfL Levels

**Figure 1:**
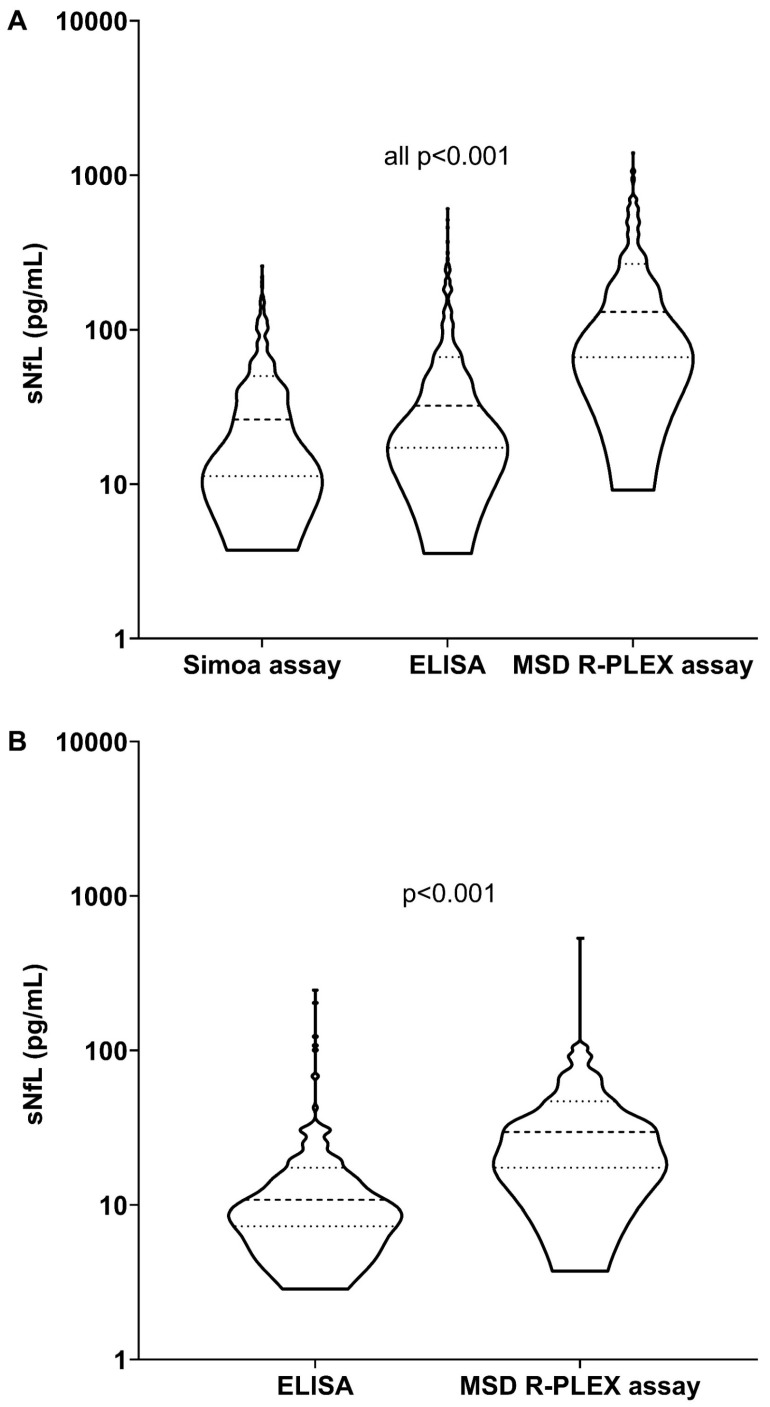
sNfL levels per assay. (**A**) sNfL levels per assay in study population; (**B**) sNfL levels per assay in healthy controls. Values are displayed as median [IQR]. Dashed line represents the median. Dotted line represents the IQR (p25–p75). ELISA: enzyme-linked immunosorbent assay; IQR: interquartile range; MSD: Meso Scale Discovery; Simoa: single-molecule array; sNfL: serum neurofilament light chain.

Median sNfL levels in the study population differed across all assays (all *p* < 0.001) and were 26.2 pg/mL, 32.2 pg/mL, and 130.5 pg/mL for the Simoa, ELISA, and MSD R-PLEX assays, respectively. In HC, median sNfL levels were 10.8 pg/mL, and 29.6 pg/mL for the ELISA and MSD R-PLEX assays, respectively (Table 1 and Figure 1A,B).

### 3.4. Passing–Bablok Regression Analysis

**Figure 2:**
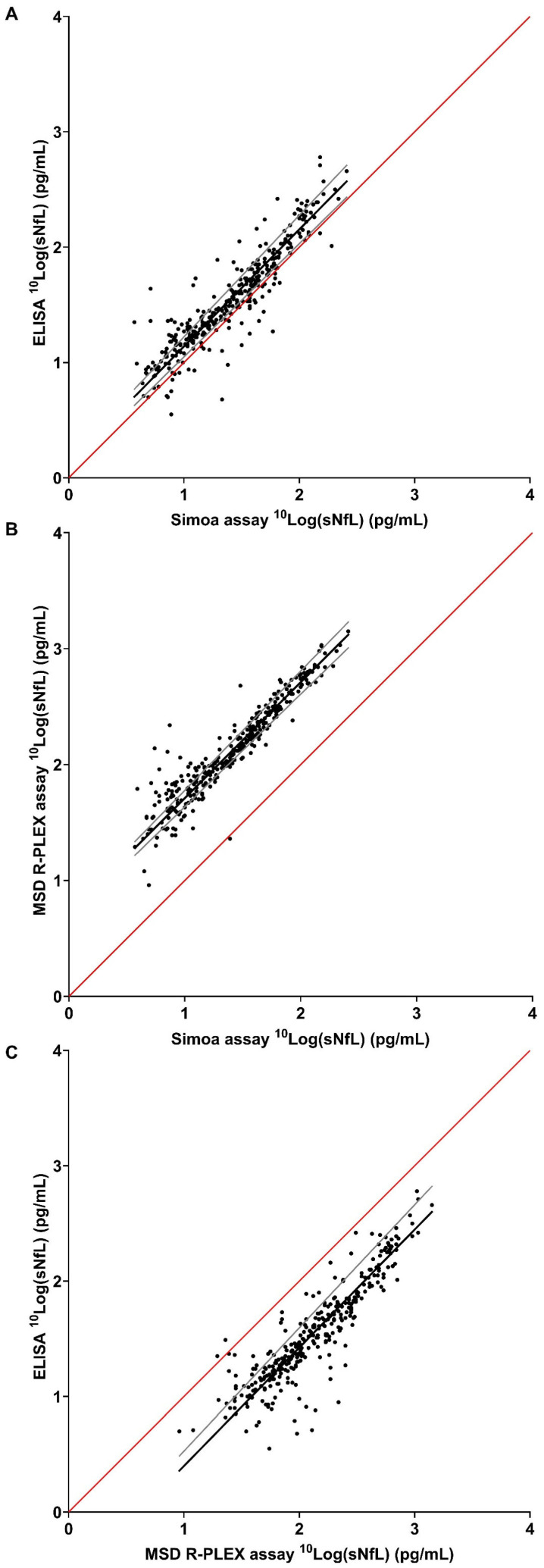
Passing–Bablok regression analysis. (**A**) Simoa assay and ELISA; (**B**) Simoa and MSD R-PLEX assays; (**C**) MSD R-PLEX assay and ELISA. Black line represents the Passing–Bablok regression line, gray line represents the 95% CI of the regression line, and the red line is the identity line (x = y). See Table 2 for corresponding values. CI: confidence interval; ELISA: enzyme-linked immunosorbent assay; MSD: Meso Scale Discovery; Simoa: single-molecule array; sNfL: serum neurofilament light chain.

The slope of the Passing–Bablok regression line for log-transformed sNfL levels was 1.01 for Simoa-ELISA, 1.00 for Simoa-MSD R-PLEX, and 1.02 for MSD R-PLEX-ELISA. Excellent correlations were found between the different assays tested (all r > 0.8) (Table 2 and Figure 2).

### 3.5. Bland–Altman Analysis and Acceptance Limits

**Figure 3:**
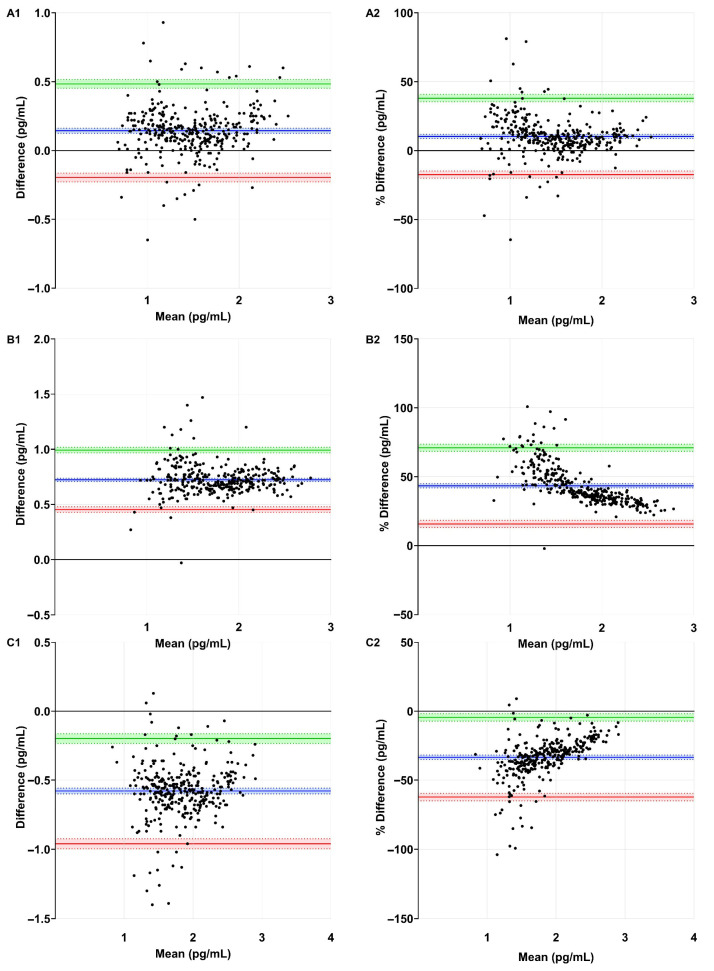
Bland–Altman analysis. (**A1**) Simoa assay and ELISA (difference); (**A2**) Simoa assay and ELISA (% difference); (**B1**) Simoa and MSD R-PLEX assays (difference); (**B2**) Simoa and MSD R-PLEX assays (% difference); (**C1**) MSD R-PLEX assay and ELISA (difference); (**C2**) MSD R-PLEX assay and ELISA (% difference). Bias with 95% CI (blue), upper limit of agreement with 95% CI (green), and lower limit of agreement with 95% CI (red) is shown. See Table 3 for corresponding values. CI: confidence interval; ELISA: enzyme-linked immunosorbent assay; MSD: Meso Scale Discovery; Simoa: single-molecule array; sNfL: serum neurofilament light chain.

Systematic bias and random error were assessed using Bland–Altman analysis, which showed a mean difference of 0.1 ± 0.2 pg/mL (10.3 ± 14.2%) for Simoa–ELISA, 0.7 ± 0.1 pg/mL (43.4 ± 14.1%) for Simoa–MSD R-PLEX, and −0.6 ± 0.2 pg/mL (−33.4 ± 14.8%) for MSD R-PLEX-ELISA (Table 3 and Figure 3(A1–C2)).

The acceptance limits were defined at ±2.1 (^10^Log[126.9]) for Simoa–ELISA, ±2.7 (^10^Log[444.9]) for Simoa–MSD R-PLEX, and ±2.7 (^10^Log[456.9]) for MSD R-PLEX-ELISA. The Bland–Altman upper and lower limits of agreement of all assay comparisons were contained within these limits (Table 3 and Figure 3(A1,B1,C1)).

### 3.6. Internal NfL Standard

To explain the differences in NfL values across assays, internal standards provided with each test were cross-measured and compared. Based on the slope of simple linear regression, the Simoa NfL internal standard yielded 2.5-fold higher values in the ELISA and 4.2-fold higher values in the MSD R-PLEX assay, while the MSD R-PLEX NfL internal standard resulted in 0.6-fold higher values in the ELISA (Figure S2).

### 3.7. Z-Scores

**Figure 4:**
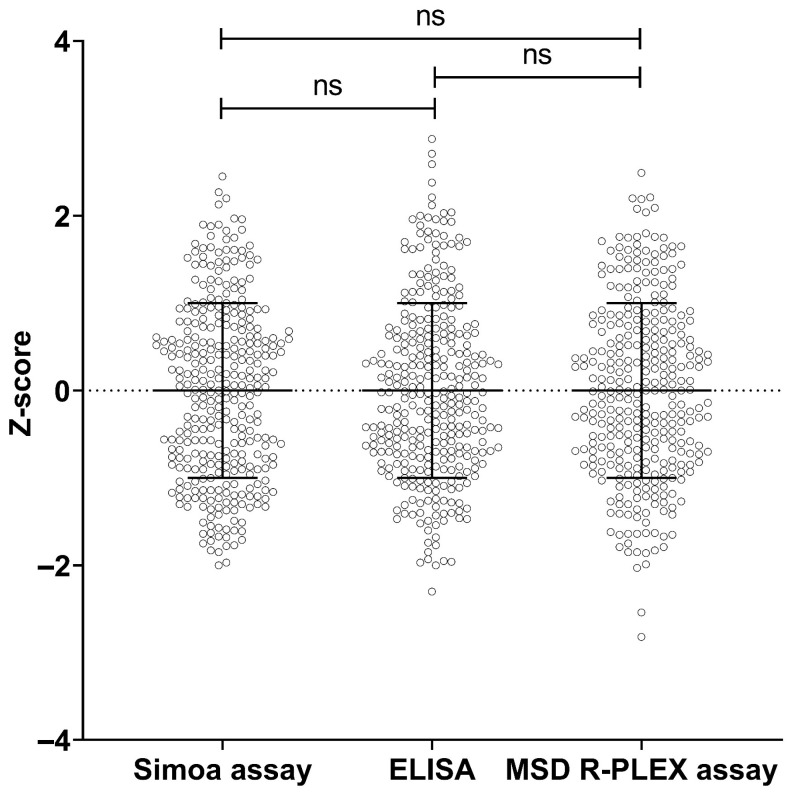
Z-scores Simoa, ELISA and MSD R-PLEX assay. The mean and standard deviation of Z-scores are shown for log-transformed sNfL. ELISA: enzyme-linked immunosorbent assay; MSD: Meso Scale Discovery; ns: not significant; Simoa: single-molecule array; sNfL: serum neurofilament light chain.

Z-scores were calculated for all samples using the mean ± SD of log-transformed sNfL levels (Table 4). The Simoa, ELISA and MSD R-PLEX assays Z-scores were comparable (all *p* = 1.0) (Figure 4). A Z-score table is provided in Table S3. An illustration of the inter-assay comparison using Z-scores from the Simoa, ELISA and MSD R-PLEX assays in longitudinal samples from individuals transitioning between disease stages and after treatment is shown in Figure S3.

### 3.8. Confirmation of Previous Study Results

In our previous study using the Simoa assay [11], we reported higher median sNfL levels in asymptomatic ATTRv patients compared to *TTR*v carriers, and in symptomatic ATTRv patients compared to *TTR*v carriers and asymptomatic ATTRv patients. In this study (*n* = 53 new samples from *n* = 14 individuals), these findings were confirmed using the ELISA, and MSD R-PLEX assays (all *p* < 0.001) (Table S4 and Figure S4).

## 4. Discussion

This study aimed to assess the performance of the ELISA and MSD R-PLEX assays to quantify sNfL levels in carriers of a pathogenic *TTR-*gene variant, allowing for the diagnostic implementation of sNfL measurement. We therefore directly compared the results of the current gold-standard Simoa assay with the ELISA and MSD R-PLEX assays. We showed (1) good agreement between the sNfL results of the ELISA and MSD R-PLEX assays compared to the Simoa assay, with some systemic and proportional differences; (2) that Z-scores enable inter-assay sNfL comparison; (3) that inter-assay differences relate to the internal NfL standards; (4) that sNfL levels increase with disease progression, consistent with our previous findings [11,13].

The Simoa assay is generally considered the current gold-standard assay for sNfL measurement [17] and the Siemens Healthineers NfL assay is the only currently available Conformité Européenne In Vitro Diagnostic Regulation approved sNfL assay [28]. The availability of different clinically validated assay formats will facilitate the widespread implementation of sNfL in clinical diagnostic practice. Moreover, a broad and unbiased diagnostic landscape requires the implemented diagnostics use of different assay formats.

In this study, the intra- and inter-assay CVs for the ELISA and MSD R-PLEX assays were all within the accepted precision limits [29]. For the Simoa assay, performance data reported by Quanterix were used [30].

Strong positive correlations were confirmed between the three assays, although several relevant differences warrant attention prior to the implementation of sNfL measurements in clinical diagnostic practice.

The Simoa–ELISA comparison indicated a small systematic bias, with the ELISA generally reporting slightly higher sNfL levels than the Simoa assay. While some proportional bias was evident, it was less pronounced than in the Simoa–MSD R-PLEX comparison. The variability observed between the Simoa and ELISA appeared to be largely random and within acceptable limits, indicating that the ELISA can be considered a viable alternative to the Simoa assay.

Compared to the Simoa assay, the MSD R-PLEX assay reported consistently 4–5-fold higher sNfL levels, as also indicated by the positive mean difference. Furthermore, there was proportional bias, with apparent overestimations by the MSD R-PLEX assay at lower sNfL levels. The bias decreased at higher sNfL levels, indicating a higher degree of agreement at higher sNfL levels. Given the lower LOD and LOQ of the Simoa assay [30] compared to the MSD R-PLEX, this pattern indicates that caution is warranted when interpreting low sNfL levels measured with the MSD R-PLEX assay relative to the more sensitive Simoa assay. Similarly to the comparison with the Simoa assay, the MSD R-PLEX assay consistently measured slightly higher sNfL levels, especially at lower sNfL levels, compared to the ELISA. Of note, the apparent overestimation by the MSD R-PLEX assay at low sNfL levels may not be clinically relevant and, as such, the MSD R-PLEX represents a suitable option for clinical diagnostic applications. However, clinicians should be aware of potential assay-dependent differences at low sNfL concentrations, especially in early or asymptomatic disease stages, where sNfL levels are typically low and small increases may indicate disease onset.

Direct measurement of the Simoa internal NfL standard in the ELISA and MSD R-PLEX assays revealed 2.5- and 4.2-fold increased values, respectively, compared to the internal standards provided with the ELISA and MSD R-PLEX assays. The MSD R-PLEX internal NfL standard revealed 0.6-fold decreased values in the ELISA. The quantitative definition of the internal standards and, as a result thereof, the definition of the level of NfL in the individual samples, may well relate to the different nature and formulation of the standards. The Simoa and ELISA use recombinant human NfL antigen as internal standard, whereas the MSD R-PLEX assay uses native NfL purified from bovine spinal cord [18,30,31]. Structural differences or post-translational modifications in the internal NfL standard, such as glycosylation, may thus be involved in the observed different test results in the respective assays, as noted previously by Hendricks et al. [32]. The actual fold differences found between the assays were approximately 1.3 (Simoa–ELISA), 5.1 (Simoa–MSD R-PLEX), and 4.2 (MSD R-PLEX-ELISA). As such, the reported difference in the detection of bovine versus recombinant human NfL is well reflected in the differences observed in our study, although other assay factors may be involved as well [17,24,25]. For example, in contrast to the antibodies used in the Simoa and ELISA, which are the same, the origin and specificity of the antibodies used in the MSD R-PLEX assay are not specified. These antibodies will likely have different antigen-binding characteristics, possibly contributing to the observed disparate sNfL values.

Other studies have also compared the Simoa, ELISA, and MSD R-PLEX assays in various cohorts [17,19,20,33,34]. Similarly to the study described here, Ulndreaj et al. reported higher sNfL levels using the MSD R-PLEX assay compared to the Simoa assay in Multiple Sclerosis patients, likely due to the differences in the antibodies and calibrators used in these assays. They observed an average 3–4-fold difference for Simoa–MSD R-PLEX [17]. Similarly, Mondesert et al. compared the Simoa, ELISA, and MSD R-PLEX assays and found, in line with our study, that the MSD R-PLEX assay reported higher sNfL levels compared to both the Simoa and ELISA. Despite the demonstration of robust overall correlation between the assays, Lin’s concordance correlation coefficient indicated agreement for Simoa–ELISA, but not for Simoa–MSD R-PLEX or MSD R-PLEX-ELISA [33]. The discrepancy between these and our findings may be partly explained by their smaller sample size and narrower measurement range. Kuhle et al. compared the Simoa, ELISA, and MSD R-PLEX assays. Good correlation was shown for the Simoa–MSD R-PLEX assays while relevant comparison of the ELISA results was impeded by the low-sensitivity ELISA format used in that study [19]. Revendova et al. and Pafiti et al. compared the Simoa and ELISA and found strong correlations. However, their analyses were limited by the lack of log-transformation, small sample sizes, and a narrower, low concentration, measurement range [20,34].

Our findings in a relatively large cohort, together with those from previous studies, underscore the importance of inter-platform calibration and detailed insight into the test characteristics, including the nature and use of internal standards. The establishment of inter-assay reference standards, and interlaboratory exchange testing, particularly with the emergence of more commercially available detection assays for NfL, is vital to the routine diagnostic clinical implementation of NfL testing [17]. The use of Z-scores provides a practical solution to allow for the direct comparison of absolute values obtained from different assays. We showed that Z-scores can be used to standardize sNfL levels, enabling inter-assay comparisons. As an example to this, Table S3 in the Supplementary Materials provides an overview of Z-scores, percentiles, and corresponding sNfL values for the Simoa, ELISA, and MSD assays. This table could be used in clinical practice to monitor both unique and longitudinally measured sNfL levels in *TTR*v carriers and ATTRv amyloidosis patients when obtained using different assays. Specifically, an sNfL value of 65 pg/mL measured using the Simoa assay corresponds to a Z-score of 1. If a subsequent follow-up measurement using the ELISA would yield an sNfL value of 343 pg/mL, this corresponds to a Z-score of 2.3, denoting a 2.3-fold increase compared to the initial measurement.

Using 53 additional samples, we confirmed our previous findings, i.e., that sNfL levels correlate with disease progression and response to treatment [11]. In addition, these findings were confirmed now using both the ELISA and MSD R-PLEX assays. Although sNfL is not a diagnostic biomarker for ATTRv amyloidosis, we show that the longitudinal monitoring of sNfL levels at the level of the individual patient provides valuable information on disease progression and response to treatment.

To date, the Simoa assay is the most commonly used research assay to study sNfL levels in carriers of a pathogenic *TTR-*gene variant [13]. To our knowledge, this is the first study comparing sNfL measurement in pathogenic *TTR-*gene variant carriers using the Simoa, ELISA and MSD R-PLEX assays. Given the diagnostic value of this biomarker for follow-up of disease progression and response to treatment, the development and definition of an inter-assay equivalence standard for other assays is warranted. In addition, further studies are needed to confirm our findings.

### Limitations

This study compared the results of sNfL measurements of the Simoa, ELISA, and MSD R-PLEX assays using retrospectively selected samples from pathogenic *TTR*-gene variant carriers from the Groningen Amyloidosis Cohort. As such, some of these samples may have undergone multiple freeze–thaw cycles for other diagnostic and/or study purposes. Given the rarity of the disease, utilizing older samples was unavoidable and allowed for a larger sample size, enhancing statistical power. We also believe that the retrospective nature of this study is unlikely to have influenced the results for several reasons. First, previous research has reported good pre-analytical stability of NfL [35]. Second, any potential influence of disease-specific treatment for amyloidosis on sNfL measurements across the assays is unlikely to have affected the comparability of the assay methods, given the consistent differences observed across the patient groups. Third, we measured sNfL levels in a total of 330 samples from 73 unique individuals. Consequently, the samples were not entirely independent. As the multiple measurements of the same individuals were taken at different time points, we believe this will not have influenced the results of comparing the assays.

Another limitation is that the samples were measured in singlicate. Despite this, Passing–Bablok and Bland–Altman analyses demonstrated good agreement between the assays, and no significant differences were observed when values were standardized using Z-scores, making sNfL levels interchangeable between assays. While this approach is generally valid, individual samples should be interpreted with caution as outliers may occasionally occur. To mitigate this limitation in diagnostic settings, we recommend performing measurements in duplicate.

## 5. Conclusions

Given some specific differences between the three different immunoassays described here, the ELISA and MSD R-PLEX assays provide suitable alternatives for the Simoa assay for measuring sNfL levels in carriers of a pathogenic *TTR-*gene variant. The differences in concentration defined by the assays directly relate to the internal standards provided with the assays. The results obtained with the different assays are interchangeable if Z-scores are used.

## Figures and Tables

**Table 1:** sNfL measurements with the Simoa, ELISA and MSD R-PLEX assays.

	Study Population (*n* = 330)			Healthy Controls (*n* = 165)	
	Simoa	ELISA	MSD R-PLEX	ELISA	MSD R-PLEX
Age (years)	53 ± 14			51 ± 17	
Male/female	144/186			62/103	
eGFR (mL/min/1.73 m^2^)	92 ± 24 (*n* = 304)			NA	
sNfL (pg/mL)	26.2 [11.3–50.1]	32.2 [17.2–66.6]	130.5 [66.4–266.3]	10.8 [7.4–17.4]	29.6 [17.8–46.6]

Data are expressed as mean ± SD when normally distributed and median [IQR] when non-normally distributed. eGFR: estimated glomerular filtration rate; ELISA: enzyme-linked immunosorbent assay; IQR: interquartile range; MSD: Meso Scale Discovery; NA: not assessed; Simoa: single-molecule array; SD: standard deviation; sNfL: serum neurofilament light chain.

**Table 2:** Passing–Bablok regression analysis in study population.

Regression	Intercept (95% CI)	Slope (95% CI)	Pearson’s r (95% CI)	*p*-Value
Simoa–ELISA	0.12 [0.07–0.17]	1.01 [0.98–1.05]	0.92 [0.90–0.93]	<0.001
Simoa–MSD R-PLEX	0.71 [0.66–0.75]	1.00 [0.97–1.03]	0.94 [0.93–0.96]	<0.001
MSD R-PLEX–ELISA	−0.62 [−0.71–−0.54]	1.02 [0.99–1.07]	0.89 [0.87–0.91]	<0.001

ELISA: enzyme-linked immunosorbent assay; MSD: Meso Scale Discovery; Simoa: single-molecule array; CI: confidence interval.

**Table 3:** Bland–Altman analysis in study population.

Comparison		Bias ± SD (95% CI)	Upper Limit of Agreement (95% CI)	Lower Limit of Agreement (95% CI)
Simoa–ELISA	pg/mL	0.1 ± 0.2 (0.1–0.2)	0.5 (0.5–0.5)	−0.2 (−0.2–−0.2)
	%	10.3 ± 14.2 (8.8–11.9)	38.1 (35.5–40.7)	−17.4 (−20.0–−14.8)
Simoa–MSD R-PLEX	pg/mL	0.7 ± 0.1 (0.7–0.7)	1.00 (1.0–1.0)	0.5 (0.4–0.5)
	%	43.4 ± 14.1 (41.9–44.9)	71.0 (68.4–73.6)	15.8 (13.2–18.4)
MSD R-PLEX—ELISA	pg/mL	−0.6 ± 0.2 (−0.6–−0.6)	−0.2 (−0.2–−0.2)	−1.0 (−1.0–−0.9)
	%	−33.4 ± 14.8 (−35.0–−31.8)	−4.5 (−7.2–−1.7)	−62.3 (−65.1–−59.6)

CI: confidence interval; ELISA: enzyme-linked immunosorbent assay; MSD: Meso Scale Discovery; Simoa: single-molecule array; SD: standard deviation.

**Table 4:** Log-transformed sNfL levels.

Assay	Simoa (*n* = 330)	ELISA (*n* = 330)	MSD R-PLEX (*n* = 330)
^10^Log sNfL	1.40 ± 0.41	1.54 ± 0.43	2.12 ± 0.41

Values are displayed as mean ± SD. ELISA: enzyme-linked immunosorbent assay; MSD: Meso Scale Discovery; SD: standard deviation; Simoa: single-molecule array; sNfL: serum neurofilament light chain.

## Data Availability

The raw data can be obtained on request from the corresponding author.

## Supplementary Materials

The following supporting information can be downloaded at: https://www.mdpi.com/article/10.3390/jcm15041584/s1, Table S1: Definitions *TTR*v carrier and (a)symptomatic ATTRv amyloidosis patient; Table S2: Genotype distribution; Table S3: Z-score table for the Simoa, ELISA, and MSD R-PLEX assays; Table S4: sNfL levels per disease stage; Figure S1: sNfL levels in healthy controls correlate with age; Figure S2: Simple linear regression analysis for internal standard NfL; Figure S3: Illustrations of inter-assay comparison using Z-scores for sNfL measurements with the Simoa, ELISA and MSD R-PLEX assays in longitudinal samples from individuals transitioning between disease stages; Figure S4: sNfL levels measured with the Simoa, ELISA and MSD R-PLEX assays in *TTR*v carriers, and (a)symptomatic ATTRv amyloidosis patients per assay; File S1: Acceptance limits. References [11,12] are cited in the Supplementary Materials.

## Abbreviations

ATTRv	hereditary transthyretin amyloid
CI	confidence interval
CSF	cerebrospinal fluid
CV	coefficient of variation
ECL	electrochemiluminescence
eGFR	estimated glomerular filtration rate
ELISA	enzyme-linked immunosorbent assay
HC	healthy controls
LOD	limit of detection
LOQ	limit of quantitation
MSD	Meso Scale Discovery
NCS	nerve conduction studies
NfL	neurofilament light chain
QST	quantitative sensory testing
SD	standard deviation
Simoa	single-molecule array
sNfL	serum neurofilament light chain
TTR	transthyretin
*TTR*v	transthyretin gene variant
UMCG	University Medical Center Groningen
