# Supplementary material for: Assessing Serum Neurofilament Light Chain in Hereditary Transthyretin Amyloidosis: Direct Comparison of Three Immunoassays
Source: J Clin Med. 2026 Feb 18;15(4):1584. doi: 10.3390/jcm15041584 (PMC12942544; doi:10.3390/jcm15041584)
Supplement: Supplementary file 1 [file jcm-15-01584-s001.zip › jcm-4123428-supplementary.pdf]

SUPPLEMENTARY TABLES

Table S1. Definitions *TTR*v carrier and (a)symptomatic ATTRv amyloidosis patient [11,12].

Disease stage	Definition
**Asymptomatic *TTR*v carrier**	Individuals with a pathogenic variant in the *TTR*-gene, but without any neurological, cardiac, gastro-intestinal and ocular signs or symptoms of disease, normal NCS and QST assessments, and the absence of amyloid in fat tissue.
**Asymptomatic ATTRv amyloidosis patient**	Patients with the presence of amyloid in fat tissue but without any neurological, cardiac, gastro-intestinal and ocular signs or symptoms of disease and normal NCS and QST assessments.
**Symptomatic ATTRv amyloidosis patient**	Patients with polyneuropathy, defined as symmetrical distal neuropathic symptoms or signs of sensory loss which had to be confirmed by NCS and/or QST assessments, with or without the presence of amyloid in fat tissue.

ATTRv: hereditary transthyretin amyloid; NCS: nerve conduction studies; QST: quantitative sensory testing; TTR: transthyretin; *TTR*v: transthyretin gene variant.

Table S2. Genotype distribution.

Genotype	Unique individuals with a pathogenic *TTR*-gene variant (n=73)
**Val30Met (p.Val50Met)**	41
**Tyr114Cys (p.Tyr134Cys)**	9
**Val71Ala (p.Val91Ala)**	6
**Glu89Lys (p.Glu109Lys)**	6
**Ser23Asn (p.Ser43Asn)**	2
**Ala45Gly (p.Ala65Gly)**	2
**Val94Ala (p.Val114Ala)**	2
**Val122Ile (p.Val142Ile)**	1
**Gly47Glu (p.Gly67Glu)**	1
**His88Arg (p.His108Arg)**	1
**Ile107Val (p.Ile127Val)**	1
**Val102del (p.Val122del)**	1

TTR: transthyretin.

Table S3. Z-score table for the Simoa, ELISA, and MSD R-PLEX assays.

		Simoa assay		ELISA		MSD R-PLEX assay	
Z-score*	Percentile	^{10}LogsNfL	sNfL	^{10}LogsNfL	sNfL	^{10}LogsNfL	sNfL
**0**	50	1,4	25,1	1,5	35,0	2,1	132,3
**0,1**	54	1,4	27,6	1,6	38,7	2,2	145,4
**0,2**	57,9	1,5	30,4	1,6	42,7	2,2	159,8
**0,3**	61,8	1,5	33,4	1,7	47,1	2,2	175,7
**0,4**	65,5	1,6	36,8	1,7	52,1	2,3	193,1
**0,5**	69,1	1,6	40,4	1,8	57,5	2,3	212,3
**0,6**	72,6	1,6	44,5	1,8	63,5	2,4	233,4
**0,7**	75,8	1,7	48,9	1,8	70,1	2,4	256,6
**0,8**	78,8	1,7	53,8	1,9	77,4	2,5	282,1
**0,9**	81,6	1,8	59,1	1,9	85,5	2,5	310,1
**1**	84,1	1,8	65,0	2,0	94,4	2,5	340,9
**1,1**	86,4	1,9	71,5	2,0	104,3	2,6	374,8
**1,2**	88,5	1,9	78,7	2,1	115,2	2,6	412,0
**1,3**	90,3	1,9	86,5	2,1	127,2	2,7	452,9
**1,4**	91,9	2,0	95,1	2,1	140,4	2,7	497,9
**1,5**	93,3	2,0	104,6	2,2	155,1	2,7	547,3
**1,6**	94,5	2,1	115,1	2,2	171,3	2,8	601,7
**1,7**	95,5	2,1	126,5	2,3	189,1	2,8	661,4
**1,8**	96,4	2,1	139,2	2,3	208,9	2,9	727,1
**1,9**	97,1	2,2	153,0	2,4	230,6	2,9	799,3
**2**	97,7	2,2	168,3	2,4	254,7	2,9	878,7
**2,1**	98,2	2,3	185,1	2,4	281,3	3,0	966,0
**2,2**	98,6	2,3	203,5	2,5	310,6	3,0	1061,9
**2,3**	98,9	2,3	223,8	2,5	343,0	3,1	1167,4
**2,4**	99,2	2,4	246,2	2,6	378,8	3,1	1283,3
**2,5**	99,4	2,4	270,7	2,6	418,3	3,1	1410,7
**2,6**	99,5	2,5	297,7	2,7	461,9	3,2	1550,9
**2,7**	99,7	2,5	327,4	2,7	510,1	3,2	1704,9
**2,8**	99,7	2,6	360,1	2,8	563,3	3,3	1874,2
**2,9**	99,8	2,6	396,0	2,8	622,1	3,3	2060,3
**3**	99,9	2,6	435,5	2,8	687,0	3,4	2265,0
**3,1**	99,9	2,7	478,9	2,9	758,6	3,4	2489,9
**3,2**	99,9	2,7	526,6	2,9	837,7	3,4	2737,2
**3,3**	100	2,8	579,2	3,0	925,1	3,5	3009,0
**3,4**	100	2,8	636,9	3,0	1021,6	3,5	3307,9
**3,5**	100	2,8	700,5	3,1	1128,2	3,6	3636,4
**3,6**	100	2,9	770,3	3,1	1245,9	3,6	3997,5
**3,7**	100	2,9	847,1	3,1	1375,8	3,6	4394,5
**3,8**	100	3,0	931,6	3,2	1519,4	3,7	4831,0
**3,9**	100	3,0	1024,5	3,2	1677,8	3,7	5310,8
**4**	100	3,1	1126,7	3,3	1852,8	3,8	5838,2
**4,1**	100	3,1	1239,1	3,3	2046,1	3,8	6418,0

**4,2**	100	3,1	1362,7	3,4	2259,6	3,8	7055,4
**4,3**	100	3,2	1498,6	3,4	2495,2	3,9	7756,1
**4,4**	100	3,2	1648,1	3,4	2755,5	3,9	8526,4
**4,5**	100	3,3	1812,4	3,5	3043,0	4,0	9373,2
**4,6**	100	3,3	1993,2	3,5	3360,4	4,0	10304,1
**4,7**	100	3,3	2192,0	3,6	3710,9	4,1	11327,5
**4,8**	100	3,4	2410,6	3,6	4098,0	4,1	12452,5
**4,9**	100	3,4	2651,0	3,7	4525,4	4,1	13689,2
**5**	100	3,5	2915,4	3,7	4997,5	4,2	15048,7

*Z-scores are calculated from log-transformed sNfL levels. Original sNfL concentrations were obtained by back-transforming using $10^{({}^{10}Log(sNfL))}$.

ELISA: enzyme-linked immunosorbent assay; MSD: Meso Scale Discovery; Simoa: single molecule array.

Table S4. sNfL levels per disease stage.

Assay	*TTR*v carriers n=73	Asymptomatic ATTRv amyloidosis patients n=49	Symptomatic ATTRv amyloidosis patients n=208
**Simoa**	9.0 [7.0–12.2]	14.7 [8.7–26.3]	41.1 [24.5–66.2]
**ELISA**	14.8 [10.3–21.7]	22.5 [13.2–37.2]	50.6 [27.0–100.8]
**MSD R-PLEX**	53.8 [37.1–70.8]	84.8 [59.1–122.8]	201.8 [108.0–385.8]

sNfL levels in pg/mL are expressed as median [IQR]. For every assay, sNfL levels were significantly different between *TTR*v carriers, asymptomatic ATTRv amyloidosis patients and symptomatic ATTRv amyloidosis patients (all $p<0.01$).

ATTRv: hereditary transthyretin amyloid; ELISA: enzyme-linked immunosorbent assay; IQR: interquartile range; MSD: Meso Scale Discovery; Simoa: single molecule array; sNfL: serum neurofilament light chain; *TTR*v: transthyretin variant.

SUPPLEMENTARY FIGURES

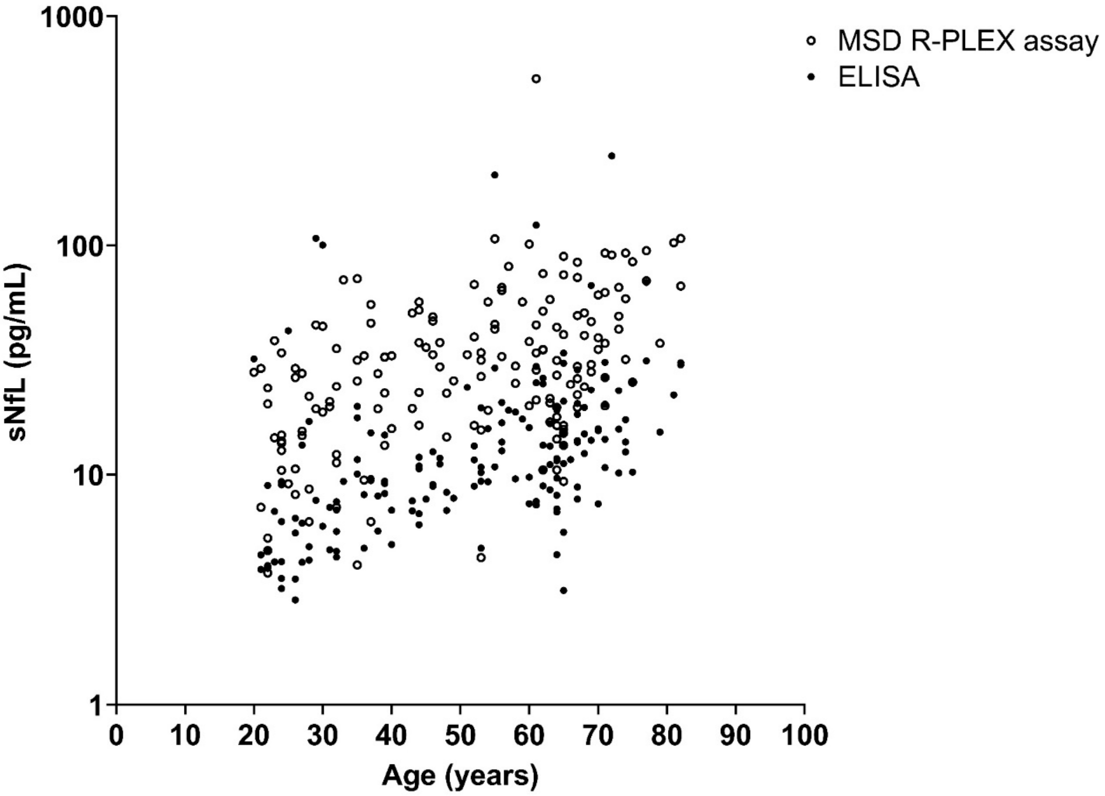

Figure S1. sNfL levels in healthy controls correlate with age.

Spearman's rho=0.56, 95% CI (0.44–0.66), p<0.001 for ELISA, and Spearman's rho=0.46, 95% CI (0.33–0.58), p<0.001 for MSD R-PLEX assay.

CI: confidence interval; ELISA: enzyme-linked immunosorbent assay; MSD: Meso Scale Discovery; sNfL: serum neurofilament light chain.

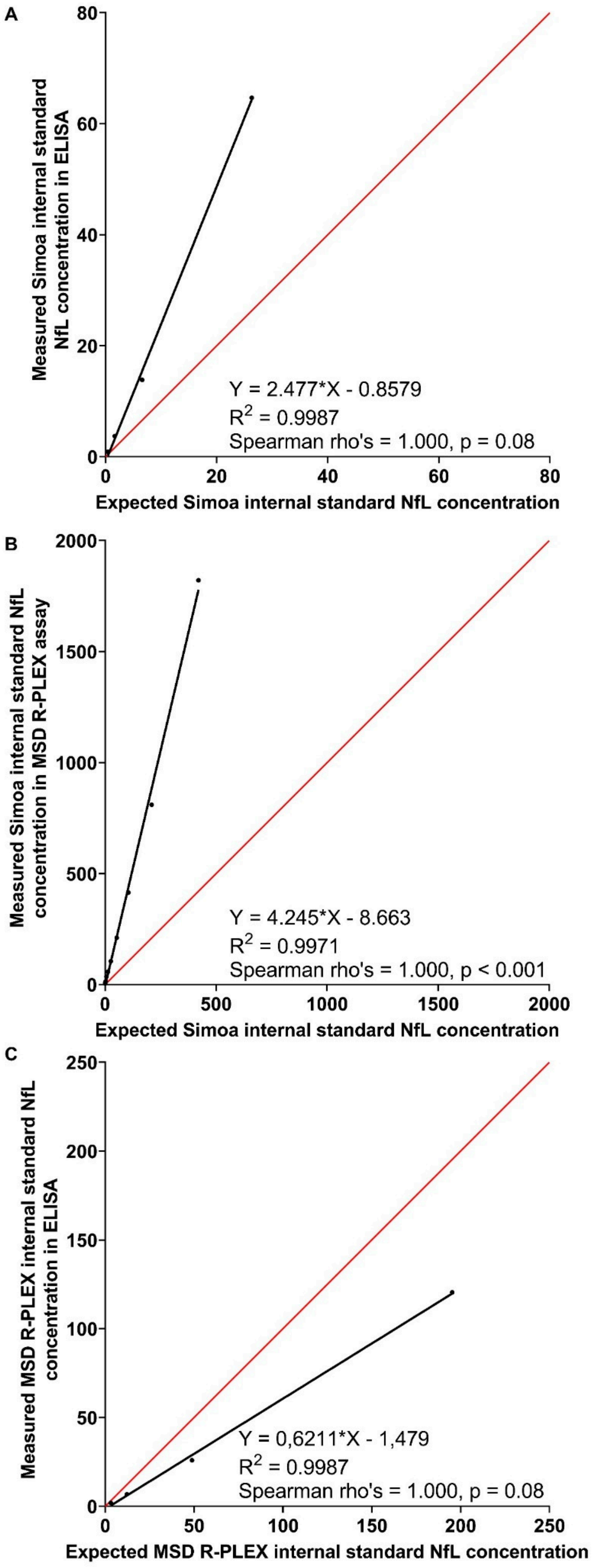
A
Measured Simoa internal standard NfL concentration in ELISA
Expected Simoa internal standard NfL concentration
Y = 2.477*X - 0.8579
R2 = 0.9987
Spearman rho's = 1.000, p = 0.08
0
20
40
60
80
B
Measured Simoa internal standard NfL concentration in MSD R-PLEX assay
Expected Simoa internal standard NfL concentration
Y = 4.245*X - 8.663
R2 = 0.9971
Spearman rho's = 1.000, p < 0.001
0
500
1000
1500
2000
C
Measured MSD R-PLEX internal standard NfL concentration in ELISA
Expected MSD R-PLEX internal standard NfL concentration
Y = 0,6211*X - 1,479
R2 = 0.9987
Spearman rho's = 1.000, p = 0.08
0
50
100
150
200
250

Figure S2. Simple linear regression analysis for internal standard NfL.

(A) Simple linear regression analysis for Simoa internal standard NfL measured in the ELISA; (**B)** Simple linear regression analysis for Simoa internal standard NfL measured in the MSD R-PLEX assay; **(C)** Simple linear regression analysis for MSD R-PLEX internal standard NfL measured in the ELISA.

Black line represents the regression line, and the red line is the identity line (x=y).

ELISA: enzyme-linked immunosorbent assay; MSD: Meso Scale Discovery; NfL: neurofilament light chain; Simoa: single molecule array.

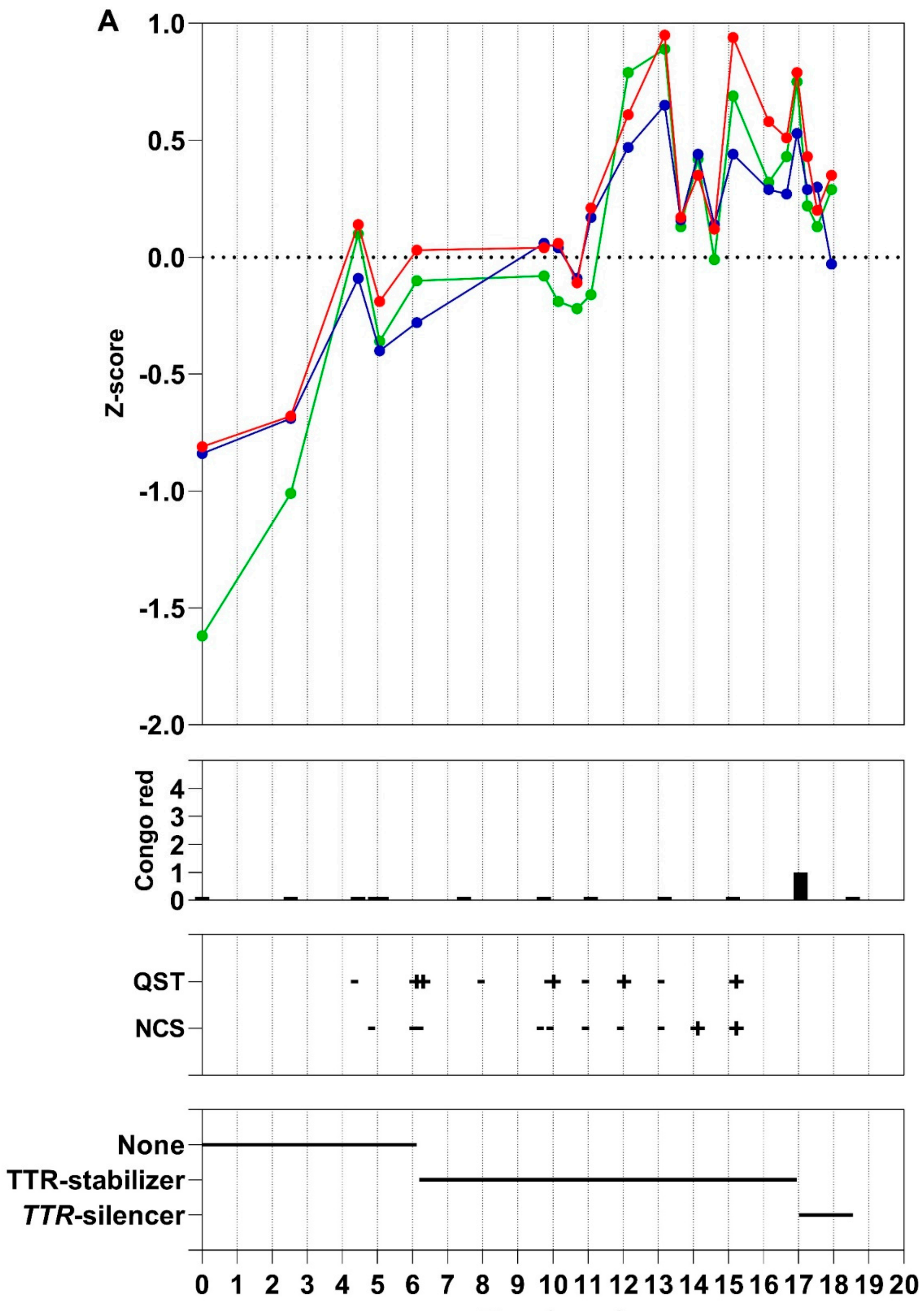
A
1.0
0.5
0.0
-0.5
-1.0
-1.5
-2.0
Z-score
Congo red
4
3
2
1
0
QST
NCS
None
TTR-stabilizer
TTR-silencer
0 1 2 3 4 5 6 7 8 9 10 11 12 13 14 15 16 17 18 19 20
Time (years)

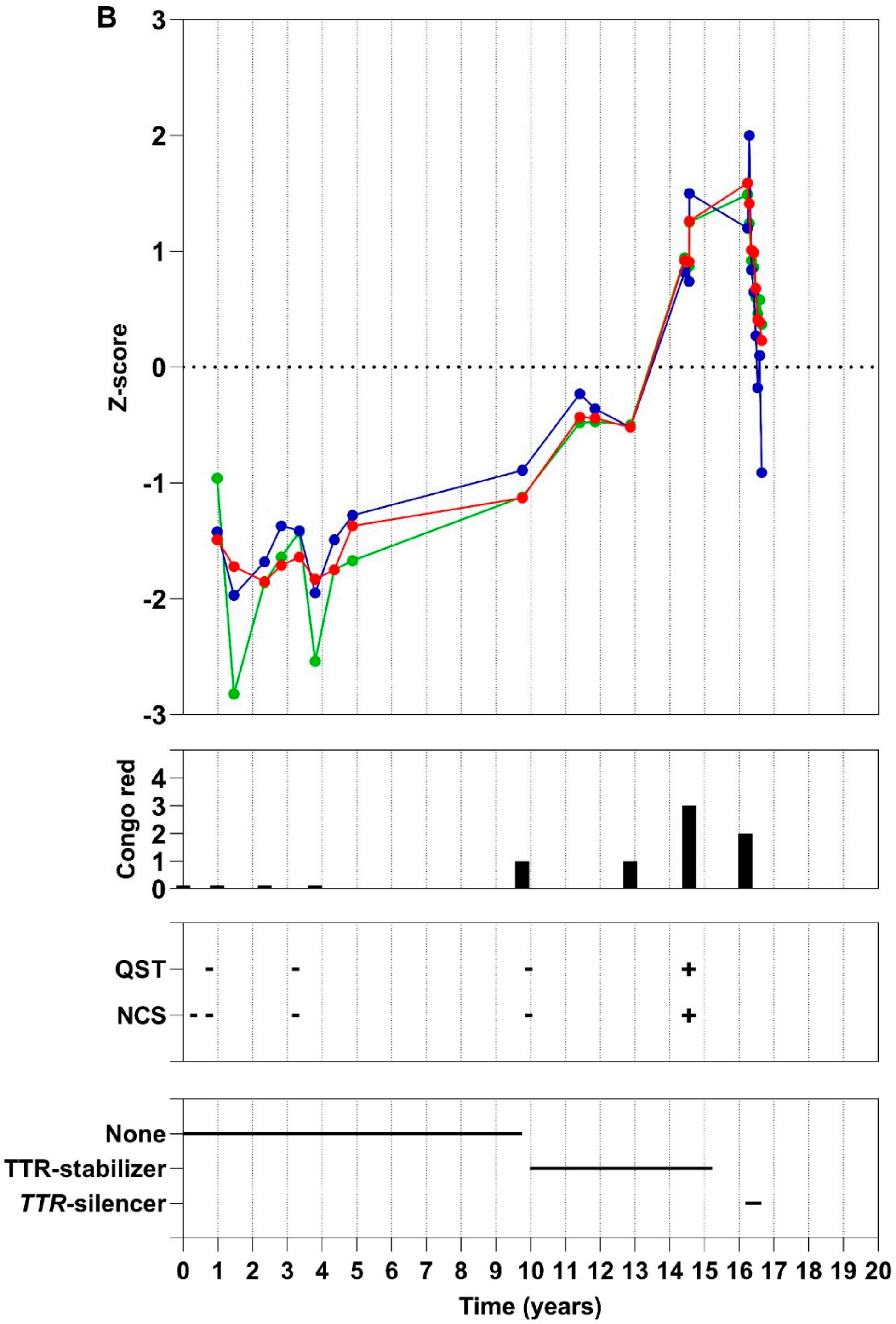
B
Z-score
3
2
1
0
-1
-2
-3
Congo red
4
3
2
1
0
QST
NCS
-
+
None
TTR-stabilizer
TTR-silencer
0 1 2 3 4 5 6 7 8 9 10 11 12 13 14 15 16 17 18 19 20
Time (years)

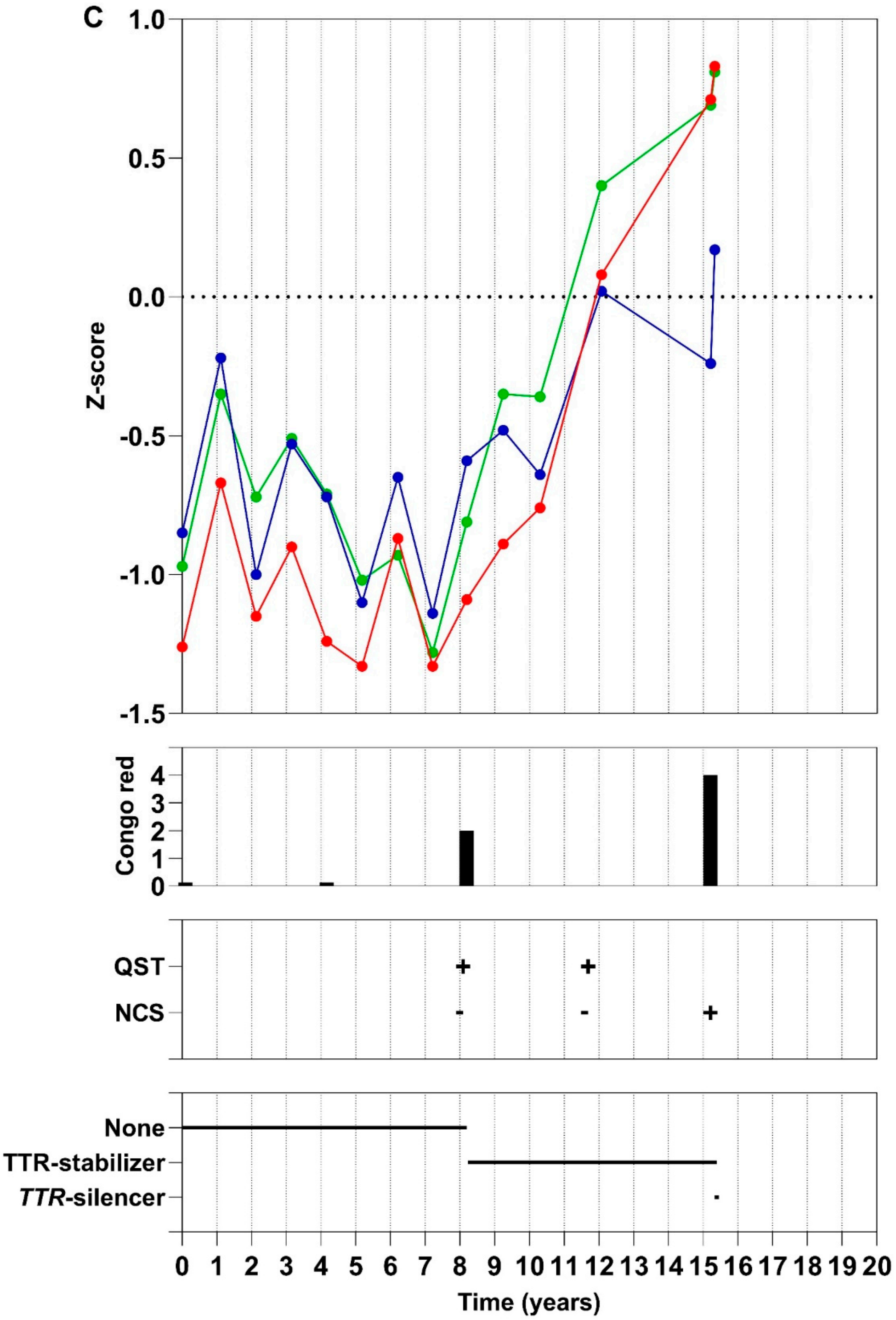

Figure S3. Illustrations of inter-assay comparison using Z-scores for sNfL measurements with the Simoa, ELISA and MSD R-PLEX assays in longitudinal samples from individuals transitioning between disease stages. **(A)** Asymptomatic *TTR*v carrier who transitioned to symptomatic ATTRv patient; **(B)** Asymptomatic *TTR*v carrier who

transitioned to symptomatic ATTRv patient; **(C)** Asymptomatic *TTR*v carrier who transitioned to symptomatic ATTRv patient.

^{10}Log(sNfL) levels measured with Simoa, ELISA and MSD R-PLEX assays were converted to a Z-score. Z-scores were calculated as (sNfL level of individual patient – mean sNfL level of study population)/ SD of study population.

Congo red positivity indicates the presence of amyloid deposits in subcutaneous fat tissue biopsy at different time points. QST and NCS results reflect the development of neuropathy. The treatment timeline shows initiation of treatment with a TTR-stabilizer and *TTR*-silencer during follow-up. For explanation about disease stages, see Table A.1.

Red line = Simoa assay; Blue line = ELISA; Green line = MSD R-PLEX assay.

ELISA: enzyme-linked immunosorbent assay; MSD: Meso Scale Discovery; NCS: nerve conduction studies; PND: polyneuropathy disability; QST: quantitative sensory testing; Simoa: single molecule array; sNfL: serum neurofilament light chain; TTR: transthyretin.

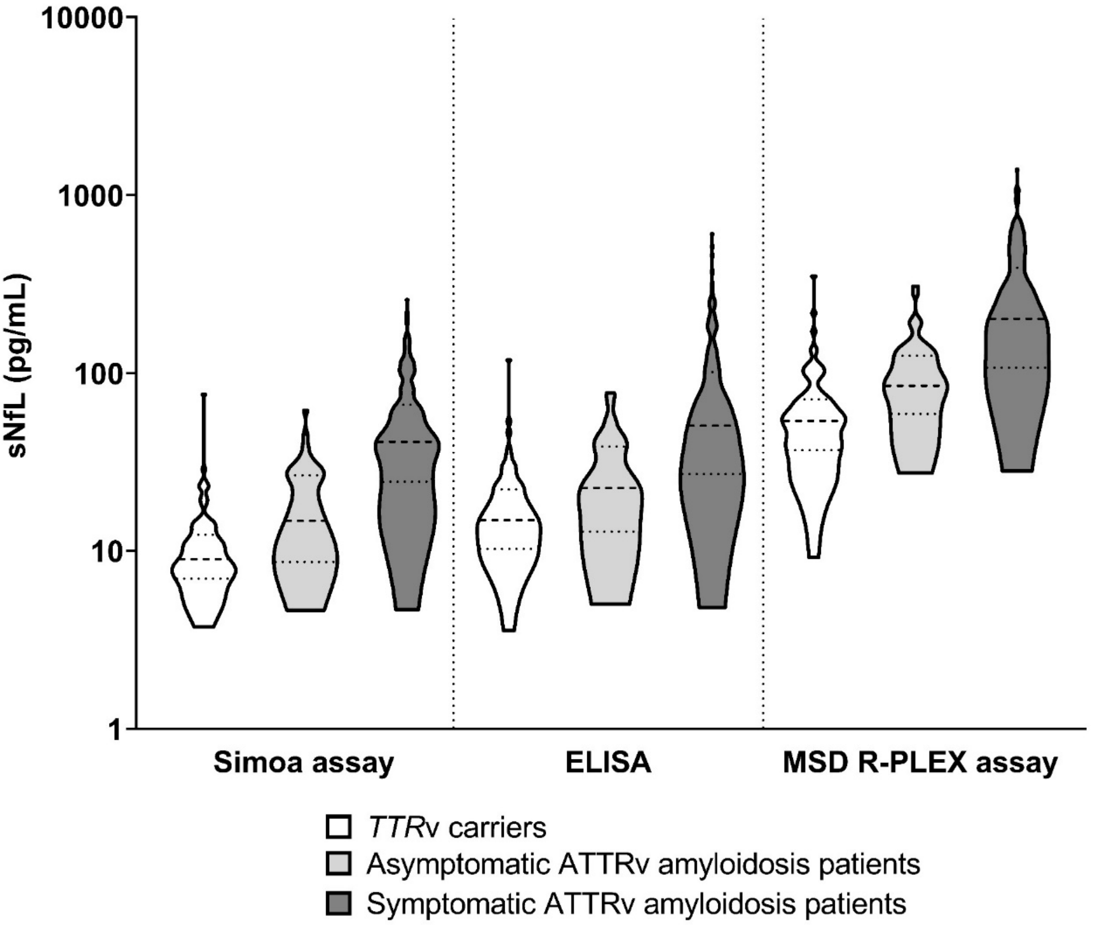

Figure S4. sNfL levels measured with the Simoa, ELISA and MSD R-PLEX assays in *TTR*v carriers, and (a)symptomatic ATTRv amyloidosis patients per assay.

Across all assays, sNfL levels were significantly different at each disease stage (all $p<0.01$). For definitions of disease stage see Table A.1.

ATTRv: hereditary transthyretin amyloid; ELISA: enzyme-linked immunosorbent assay; MSD: Meso Scale Discovery; NfL: neurofilament light chain; Simoa: single molecule array; *TTR*v: transthyretin gene variant.

File S1. Acceptance limits.

The acceptance limits for easy assay comparison were calculated as follows: $acceptance\ limit_{absolute} = acceptance\ limit_{percentage} * mean$,

with $acceptance\ limit_{percentage} = \sqrt{\frac{intra\text{−}assay\ CV\ (\%)_A^2}{n} + \frac{intra\text{−}assay\ CV\ (\%)_B^2}{n}}$, and $mean = \frac{mean\ assay_A + mean\ assay_B}{2}$, in which A and B correspond to the assay method (*i.e.* Simoa, ELISA, or MSD R-PLEX assay) and n to the number of samples used for calculating intra-assay CV. The absolute acceptance limit was log-transformed with the formula: $^{10}Log(acceptance\ limit_{absolute})$.
